# The body mass index-glucose index as a new tool for early detection of the risk of dysglycemia in patients with hypertension and obstructive sleep apnea

**DOI:** 10.1186/s12902-026-02226-w

**Published:** 2026-03-19

**Authors:** Wenbo Yang, Xintian Cai, Mulalibieke Heizhati, Qing Zhu, Xiaoguang Yao, Wen Wen, Di Shen, Junli Hu, Jing Hong, Nanfang Li

**Affiliations:** Xinjiang Hypertension Institute, NHC Key Laboratory of Hypertension Clinical Research, Key Laboratory of Xinjiang Uygur Autonomous Region “Hypertension Research Laboratory”, Xinjiang Clinical Medical Research Center for Hypertension (Cardio-Cerebrovascular) Diseases, Hypertension Center of People’s Hospital of Xinjiang Uygur Autonomous Region, NO. 91 TianChi Road, Urumqi, Xinjiang 830001 China

**Keywords:** Hypertension, Obstructive sleep apnea, Dysglycemia, Body mass index-glucose index, Retrospective cohort

## Abstract

**Purpose:**

Currently, there is a lack of early biomarkers to identify the risk of dysglycemia in patients with concurrent hypertension and obstructive sleep apnea (OSA). The aim of our study is to evaluate the efficacy of the recently proposed Body Mass Index (BMI)-Glucose (ByG) index in identifying the risk of dysglycemia in patients with hypertension and OSA.

**Methods:**

A retrospective cohort study of 1,759 adults with hypertension and OSA from the Urumqi Research on Sleep Apnea and Hypertension study (UROSAH) was conducted. Cox proportional hazards models were used to assess the associations between the ByG index and new-onset dysglycemia, diabetes, and prediabetes. Time-dependent receiver operating characteristic (ROC) curves were constructed to compare the efficacy of the ByG index with traditional insulin resistance indicators.

**Results:**

During a median follow-up of 7.25 years, 212 cases of dysglycemia (157 diabetes, 55 prediabetes) were identified. Participants in the highest ByG tertile had a significantly increased risk of dysglycemia (HR 3.20; 95% CI: 2.13–4.80), diabetes (HR 3.38; 95% CI: 2.02–5.66), and prediabetes (HR 2.95; 95% CI: 1.44–6.06) compared to the lowest tertile, after full adjustment. Time-dependent ROC showed the ByG index was more discriminative in predicting dysglycemia (including diabetes and prediabetes) events at 3, 5 and 7 years compared to BMI, TyG, TyG-BMI indices, waist circumference, and waist-to-height ratio.

**Conclusion:**

The ByG index serves as an independent predictor of dysglycemia, encompassing diabetes and prediabetes, among patients with hypertension and OSA. As a simple, accessible, and reliable tool for early risk stratification, it demonstrates application potential but requires validation through prospective studies.

**Trial registration:**

Not applicable.

**Supplementary Information:**

The online version contains supplementary material available at 10.1186/s12902-026-02226-w.

## Introduction

Diabetes affects approximately 530 million adults worldwide with a prevalence of 10.5% in people aged 20–79 years, posing a significant burden on global health [1–3]. Remarkably, hypertension and obstructive sleep apnea (OSA) significantly increase the risk of developing diabetes [4–6]. Patients with OSA have a 1.4 times higher risk of developing diabetes, whereas those with hypertension have a 1.61 times higher risk [7, 8]. The prevalence of diabetes is notably higher among patients with both hypertension and OSA. Additionally, these individuals face a greater burden of cardiovascular disease after developing diabetes [9–11]. Therefore, to optimise diabetes management strategies, it is essential to find reliable and easy-to-use biomarkers for early identification of the risk of dysglycemia in this high-risk population. Nonetheless, few studies focus on predicting the risk of dysglycemia in patients with both hypertension and OSA, and there are currently no simple and convenient biomarkers available for the early identification of people at high risk of dysglycemia.

Insulin resistance is widely regarded as an early detection indicator of the development of dysglycemia and also is considered to be one of the main pathological mechanisms underlying the development of dysglycemia in patients with hypertension and OSA [12–14]. The hyperinsulin glucose clamp is the gold standard for detecting insulin resistance [15]; however, its complexity, time consumption, and invasiveness limit its widespread use. Recently, a new, simple, and promising indicator for evaluating insulin resistance has recently been proposed and is known as Body mass index (BMI)-glucose (ByG) index [16]. Defined as Ln [1/2 BMI (kg/m²) × fasting plasma glucose (FPG) (mg/dL)], the ByG index demonstrated good predictive value in the general population and outperformed traditional markers like BMI, triglyceride glucose (TyG) and triglyceride glucose-BMI (TyG-BMI) in predicting diabetes risk [16]. Given its simplicity, convenience, cost-effectiveness, and predictive value, coupled with the lack of early identification indicators for dysglycemia in patients with OSA and hypertension, it is imperative to study the relationship between these factors.

Therefore, this study utilized data from the Urumqi Research on Sleep Apnea and Hypertension study (UROSAH) to explore the association between the ByG index and dysglycemia, including diabetes and prediabetes, in individuals with hypertension and OSA. In addition, we also compared the diagnostic capabilities of the ByG index with traditional classic indicators BMI, TyG, TyG-BMI indices, waist circumference (WC), and waist-to-height ratio (WHtR) to identify dysglycemia at different time points.

## Materials and methods

### Study population

**Fig. 1:**
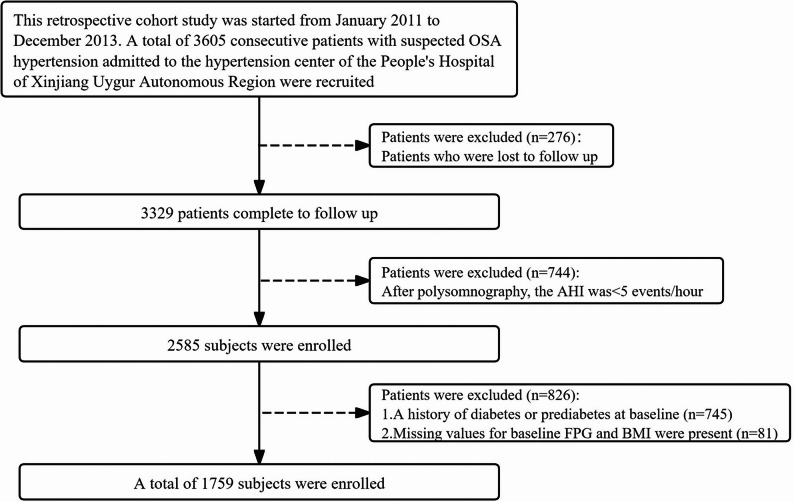
Flow chart of selected participants

This study used data from the UROSAH cohort, which has been previously detailed [17]. Briefly, the UROSAH cohort was a single-center observational study conducted at the Hypertension Center of Xinjiang Uygur Autonomous Region People’s Hospital, a provincial tertiary care hospital. The aim of the UROSAH study was to investigate the association between OSA and long-term cardiovascular outcomes in patients with hypertension. This retrospective cohort study enrolled 3605 consecutive hypertensive patients with suspected OSA. In this analysis, 744 participants were excluded because their apnea-hypopnea index (AHI) was less than 5 events per hour, as measured by polysomnography. Moreover, 826 individuals excluded for the following reasons: presence of diabetes or prediabetes at baseline (*n* = 745), or missing baseline values for FPG or BMI (*n* = 81). A total of 1,759 participants were included in this study (Fig. 1).

### Ethical approval

Approval for this research was granted by the Ethics Committee at the People’s Hospital in the Xinjiang Uygur Autonomous Region (reference: 2019030662). The research followed the ethical principles specified in the Declaration of Helsinki. Consent was obtained from every participant, ensuring that the collection and use of data were conducted ethically throughout the duration of the study.

### Data collection

Experienced researchers systematically collected baseline data including participant demographics, medical history, and laboratory findings. Height and weight were measured using standardized protocols. Waist circumference (WC) was measured with a non-stretchable tape at the horizontal midpoint between the iliac crest and the lowest rib. BMI was calculated the equation: body weight in kilograms divided by the square of height in meters (kg/m²). Skilled professionals recorded blood pressure measurements using standardized methods referenced in previous studies [17]. Healthcare professionals compiled each participant’s medical history including demographic details, lifestyle factors, medication use, and previous health conditions.

Laboratory analysis encompassed a range of blood tests, including FPG, low-density lipoprotein, triglyceride, high-density lipoprotein (HDL), total cholesterol, aspartate aminotransferase, alanine aminotransferase, and serum creatinine. These tests were conducted on peripheral venous blood samples collected after a 12-hour fasting period. The glomerular filtration rate was determined through the application of the Chronic Kidney Disease Epidemiology Collaboration-derived equations [18].

Additionally, all participants underwent overnight polysomnography in a controlled laboratory environment, following the detailed protocols provided in the Supplemental material.

### Definitions at baseline

The ByG index was determined through the equation ByG = Ln [1/2 × BMI (kg/m²) × FPG (mg/dL)] [16]. TyG = Ln [(FPG (mg/dL) × triglyceride (mg/dL) / 2]. TyG-BMI = BMI × TyG [16]. The WHtR was calculated as waist circumference (cm) divided by height (cm). In alignment with the Chinese health industry standard WS/T 428–2013, obesity was classified as a BMI of 28 kg/m² or higher, overweight was defined as a BMI ranging from 24 to 28 kg/m². Hypertension was identified based on the 2010 Chinese Hypertension Prevention and Treatment Guidelines, which specify a resting blood pressure of 140/90 mmHg or higher or the current use of antihypertensive medications. OSA diagnosis was established when the AHI exceeded five events per hour. The severity of OSA was categorized as mild for an AHI between 5 and less than 15 events per hour, moderate for an AHI of 15 to less than 30 events per hour, and severe for an AHI exceeding 30 events per hour [19]. Adherence to regular continuous positive airway pressure (CPAP) therapy was defined as using CPAP for more than 70% of nights and at least four hours per night during the follow-up period [20, 21]. Smoking and alcohol consumption were grouped into two categories: “Current” for individuals who currently smoke or consume alcohol or who ceased within the past year, and “Never or Former” for those who have never engaged in these habits or who discontinued them more than one year prior.

### Follow-up and outcome

A comprehensive follow-up process was implemented, encompassing outpatient visits, inpatient medical records, and telephone interviews. Participants were monitored for the development of dysglycemia, a term encompassing both diabetes and prediabetes, until the conclusion of the follow-up in January 2021. Diabetes was diagnosed when fasting plasma glucose levels were equal to or greater than 7.0 mmol/L and/or 2-hour plasma glucose levels reached or exceeded 11.1 mmol/L during an oral glucose tolerance test, or if an individual was utilizing antidiabetic medications [22]. Prediabetes was defined as two conditions: impaired fasting glucose and impaired glucose tolerance. Impaired fasting glucose was defined as an FPG level between 6.1 and 6.9 mmol/L, with a 2-hour postprandial glucose level of less than 7.8 mmol/L. Impaired glucose tolerance was defined as a 2-hour postprandial glucose level between 7.8 and 11.0 mmol/L ^22^. All events were verified using medical records and confirmed by the clinical event committee in accordance with the protocols detailed in prior studies [17, 23].

### Statistical analysis

Descriptive analyses were performed to characterize the dataset, with continuous variables presented as mean values accompanied by their standard deviations, while categorical variables were reported as both frequency counts and corresponding percentages. Comparative analyses of participant characteristics across ByG tertiles were conducted using appropriate statistical tests, including the one-way analysis of variance, Fisher’s exact test, Kruskal-Wallis test, and chi-square tests.

Visualize the unadjusted cumulative risk using Kaplan-Meier analysis and determine significance using the Log-rank test. Multicollinearity among predictor variables was assessed through variance inflation factor calculations, with variables exhibiting variance inflation factor values greater than 5 being excluded from subsequent analyses (Supplemental Table 1). To evaluate the association between ByG (analyzed both continuously and by tertiles) and the new-onset of dysglycemia, a Cox proportional hazards regression model was implemented, providing hazard ratios (HR) with corresponding 95% confidence intervals (CI) for diabetes, prediabetes, and overall dysglycemia outcomes. Three analytical models were constructed: The initial model (Model 1) incorporated demographic factors including age, sex and ethnicity. The subsequent model (Model 2) extended the first model by incorporating alcohol consumption, smoking status, diastolic and systolic blood pressure, and coronary heart disease and stroke history. The most comprehensive model (Model 3) further augmented Model 2 by integrating biochemical markers (aminotransferase levels, estimated glomerular filtration rate, HDL, and triglycerides), pharmacological interventions (angiotensin receptor blockers, ACE inhibitors, diuretics, beta-blockers, calcium channel blockers, and statins), sleep-related parameters (AHI, nadir oxygen saturation, mean oxygen saturation), and therapeutic interventions (continuous positive airway pressure treatment). To assess potential trends, the median values of each tertile were assigned to participants and analyzed as continuous variables within the Cox proportional hazards regression framework.

In order to explore the potential for nonlinear associations between the ByG index and dysglycemia, diabetes and prediabetes, we performed restricted cubic spline analyses using Cox regression models. These analyses were conducted after the correction of all confounders in model 3. A range of three to seven node configurations were evaluated, and the configuration yielding the lowest Akaike Information Criterion value was selected for the final analysis. For dysglycemia and diabetes, four nodes were positioned at the 5th, 35th, 65th, and 95th percentiles, while for prediabetes, five nodes were placed at the 5th, 28th, 50th, 72nd, and 95th percentiles. Restricted cubic spline analysis identified an inflection point that segmented the ByG index into two parts, thus allowing the modeling of distinct association patterns between the ByG index and outcomes using segmented Cox regression. In addition, to compare the efficacy of the ByG index with traditional classic insulin resistance indicators (including BMI, TyG, TyG-BMI, WC, and WHtR) for diagnosing dysglycemia, diabetes, and prediabetes at different time points, we constructed time-dependent receiver operating characteristic (ROC) curves and calculated the area under the ROC curve (AUC).

Multiple sensitivity analyses were performed to verify the robustness of our findings. Initially, individuals who were current smokers or drinkers were excluded to assess the impact of residual confounding factors. Secondly, participants who received regular OSA treatment were excluded to evaluate the possible treatment-related confounding factors. Thirdly, a one-year lag analysis was conducted, which excluded patients who experienced dysglycemia during the first year of follow-up. Fourthly, we separately excluded individuals using diuretics and those with a history of stroke at baseline due to the potential effects of diuretics on glucose metabolism and the baseline differences in stroke rates between the groups. Stratified and interaction analyses were also carried out based on several key factors: age (< 45 or ≥ 45 years), gender, drinking status, smoking status, AHI (< 15, 15–30, or ≥ 30 events/h), BMI (< 28 or ≥ 28 kg/m²), diastolic blood pressure (DBP) (< 90 or ≥ 90 mmHg), systolic blood pressure (SBP) (< 140 or ≥ 140 mmHg), angiotensin-converting enzyme inhibitors(ACEIs) /angiotensin receptor blockers (ARBs) use, statin use, and baseline stroke.

All statistical tests were two-sided, with a significance threshold of *P* < 0.05 (two-sided). Analyses were carried out using the statistical software R version 4.2.2.

## Results

### Baseline characteristics

The baseline analysis involved 1,759 participants, 69.0% of whom were male, with an average age of 48.79 years. Participants were stratified into three groups based on the ByG index: tertile 1 (≤ 7.01), tertile 2 (7.01 < ByG ≤ 7.16), and tertile 3 (≥ 7.16).

Participants in the highest ByG tertile were generally younger, had a lower incidence of baseline stroke, and had a higher likelihood of being male. Additionally, this group showed a higher prevalence of obesity and an increased likelihood of alcohol consumption.

Clinically, participants in the high ByG group exhibited slightly elevated levels of aminotransferase. As expected, triglyceride and FPG levels rose with higher ByG tertiles, HDL levels significantly decreased. Regarding medication use, the prevalence of ACEI and CCB was higher in the high ByG group, while statin use was consistent across all groups. Additionally, participants with a higher ByG index exhibited more severe OSA, indicated by higher AHI values and lower nocturnal minimum and mean oxygen saturation levels (Table 1).

**Table 1:** Baseline characteristics of participants by ByG index tertiles

Characteristics	Overall	ByG Tertile 1	ByG Tertile 2	ByG Tertile 3	*P*-value
		(< 7.01)	(≥ 7.01 to < 7.16)	(≥ 7.16)	
Participants, n (%)	1759	586	587	586	
Demographic characteristics					
Age, years	48.79 ± 10.68	49.95 ± 11.05	48.83 ± 10.63	47.58 ± 10.24	< 0.001
Male, n (%)	1214 (69.0%)	368 (62.8%)	426 (72.6%)	420 (71.7%)	< 0.001
Ethnicity, n (%)					< 0.001
Han	1,134 (64.5%)	425 (72.5%)	391 (66.6%)	318 (54.3%)	
Uyghur	385 (21.9%)	88 (15.0%)	106 (18.1%)	191 (32.6%)	
Other	240 (13.6%)	73 (12.5%)	90 (15.3%)	77 (13.1%)	
Current smokers, n (%)	769 (43.7%)	237 (40.4%)	275 (46.8%)	257 (43.9%)	0.086
Current drinkers, n (%)	709 (40.3%)	200 (34.1%)	254 (43.3%)	255 (43.5%)	< 0.001
BMI, kg/m^2^	28.06 ± 3.71	25.17 ± 2.50	27.99 ± 2.51	31.01 ± 3.42	0.002
SBP, mmHg	139.56 ± 19.75	139.61 ± 19.77	138.58 ± 19.28	140.48 ± 20.19	0.257
DBP, mmHg	92.01 ± 14.01	91.52 ± 13.91	91.51 ± 14.00	93.00 ± 14.11	0.113
Baseline CHD, n (%)	176(10.0%)	51(8.7%)	63(10.7%)	62(10.0%)	0.435
Baseline Stroke, n (%)	365(20.8%)	138(23.5%)	131(22.3%)	96(16.4%)	0.005
Clinical laboratory measurements					
AST, U/L	22.26 ± 15.57	22.84 ± 23.29	21.11 ± 8.02	22.84 ± 11.06	0.093
ALT, U/L	27.86 ± 20.17	25.98 ± 23.01	26.32 ± 16.42	31.26 ± 20.84	< 0.001
eGFR, ml/min/1.73 m^2^	95.59 ± 20.89	94.81 ± 20.48	95.49 ± 21.32	96.47 ± 20.86	0.394
TC, mmol/L	4.52 ± 1.11	4.52 ± 1.19	4.47 ± 1.05	4.58 ± 1.10	0.260
TG, mmol/L	2.02 ± 1.41	1.82 ± 1.33	2.03 ± 1.39	2.20 ± 1.50	< 0.001
HDL-C, mmol/L	1.12 ± 0.30	1.19 ± 0.32	1.10 ± 0.27	1.07 ± 0.27	< 0.001
LDL-C, mmol/L	2.66 ± 0.78	2.65 ± 0.81	2.63 ± 0.77	2.68 ± 0.76	0.323
FPG, mmol/L	4.80 ± 0.62	4.35 ± 0.50	4.79 ± 0.40	5.27 ± 0.58	< 0.001
ByG	7.08 ± 0.19	6.88 ± 0.14	7.09 ± 0.04	7.28 ± 0.10	< 0.001
Prescribed medication, n (%)					
ACEIs/ARBs users, n (%)	843 (47.9%)	252 (43.0%)	281 (47.9%)	310 (52.9%)	0.003
β-blockers users, n (%)	171 (9.7%)	52 (8.9%)	62 (10.6%)	57 (9.7%)	0.621
CCBs users, n (%)	1285 (73.1%)	395 (67.4%)	437 (74.4%)	453 (77.3%)	< 0.001
Diuretics users, n (%)	303 (17.2%)	94 (16.0%)	105 (17.9%)	104 (17.7%)	0.648
Statins users, n (%)	942 (53.6%)	313 (53.4%)	324 (55.2%)	305 (52.0%)	0.806
PSG parameters					
AHI, events/h	23.48 ± 18.11	20.05 ± 15.23	23.21 ± 17.12	27.18 ± 20.83	< 0.001
Moderate-severe OSA, n (%)	1062 (60.4%)	317 (51.1%)	357 (60.8%)	388 (66.2%)	< 0.001
Nadir SaO_2_,%	78.25 ± 8.69	79.97 ± 7.45	78.64 ± 7.60	76.16 ± 10.29	< 0.001
Mean SaO_2_,%	91.97 ± 3.37	92.59 ± 2.60	91.85 ± 4.17	91.46 ± 3.05	< 0.001
Regular CPAP treatment, n (%)	39 (2.2%)	8 (1.4%)	15 (2.6%)	16 (2.7%)	0.225

Values of continuous variables are expressed as medians (twenty-fifth percentile - seventy-fifth percentile) or means (standard deviation). Categorical variables are expressed as no. (%)

ALT, alanine aminotransferase; AST, aspartate aminotransferase; BMI, body mass index; DBP, diastolic blood pressure; FPG, fasting plasma glucose; SBP, systolic blood pressure; HDL-C, high-density lipoprotein cholesterol; CHD, coronary heart disease; eGFR, estimated glomerular filtration rate; LDL-C, low density lipoprotein cholesterol; TC, total cholesterol; AHI, apnea hypopnea index; TG, triglyceride; ACEIs, angiotensin-converting enzyme inhibitors; ARBs, angiotensin receptor blockers; CCBs, calcium channel blockers; PSG, polysomnography; OSA, obstructive sleep apnea; CPAP, continuous positive airway pressure; SaO_2_, oxygen saturation; ByG, body mass index-glucose index

### Association of ByG index with the risk of new-onset dysglycemia, diabetes, and prediabetes

**Fig. 2:**
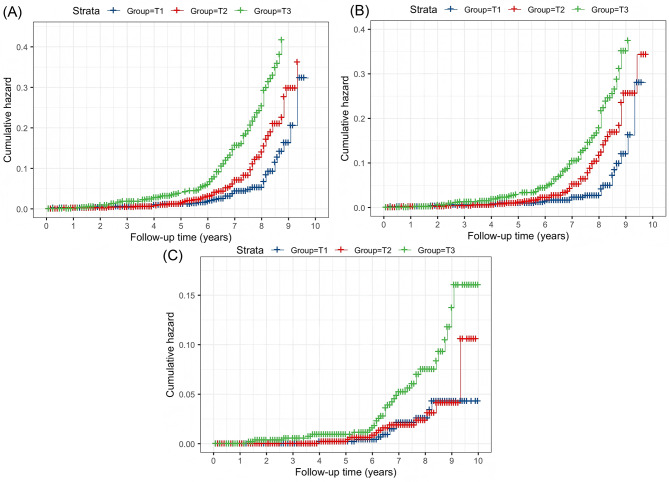
Cumulative hazard curves for new-onset of dysglycemia (**A**), diabetes (**B**), and prediabetes (**C**), stratified by ByG index tertiles

Over a median follow-up period of 7.25 years, that equates to 11,662.1 person-years, 212 new cases of dysglycemia were observed, included 157 cases of diabetes (8.93%) and 55 cases of prediabetes (3.13%). The analysis of cumulative risk curves indicated that participants with higher ByG indices experienced a significant increase in the incidence of dysglycemia, diabetes, and prediabetes (log-rank *P* < 0.001; Fig. 2A-C).

Table 2 presents the relationship between ByG levels and the development of dysglycemia, diabetes, and prediabetes. When ByG was analyzed as a continuous variable, each 1 SD increase in ByG was associated with a 56% elevated risk of incident dysglycemia (95% CI: 1.34–1.80), a 64% higher risk of incident diabetes (95% CI: 1.42–1.97), and a 39% increased risk of incident prediabetes (95% CI: 1.04–1.84). In the tertile-based analysis, participants in the highest tertile exhibited significantly higher risks of dysglycemia (HR: 3.20; 95% CI: 2.13–4.80), diabetes (HR: 3.38; 95% CI: 2.02–5.66), and prediabetes (HR: 2.95; 95% CI: 1.44–6.06) compared to those in the lowest tertile, after adjusting for potential confounders (Model 3). The middle tertile also demonstrated a significantly elevated risk for these outcomes relative to the lowest tertile. Furthermore, a trend test indicated a dose-dependent increase in risk across the tertile groups.

**Table 2:** Hazard ratios (95% CI) of dysglycemia, diabetes, and prediabetes stratified by ByG index

Exposure	Model 1		Model 2		Model 3	
	(HR, 95% CI)	*P*-value	(HR, 95% CI)	*P*-value	(HR, 95% CI)	*P*-value
**Dysglycemia**						
Per SD increment	1.57 (1.36, 1.80)	< 0.001	1.61 (1.40, 1.85)	< 0.001	1.56 (1.34, 1.80)	< 0.001
ByG Tertiles						
T1	Reference		Reference		Reference	
T2	1.70 (1.10, 2.62)	0.017	1.69 (1.09, 2.61)	0.018	1.60 (1.03, 2.49)	0.037
T3	3.32 (2.24, 4.91)	< 0.001	3.49 (2.35, 5.18)	< 0.001	3.20 (2.13, 4.80)	< 0.001
P for trend		< 0.001		< 0.001		< 0.001
**Diabetes**						
Per SD increment	1.65 (1.40, 1.94)	< 0.001	1.68 (1.42, 1.97)	< 0.001	1.64 (1.38, 1.95)	< 0.001
ByG Tertiles						
T1	Reference		Reference		Reference	
T2	2.16 (1.31, 3.57)	0.003	2.19 (1.32, 3.61)	0.002	2.05 (1.20, 3.48)	0.008
T3	3.70 (2.31, 5.91)	< 0.001	3.77 (2.35, 6.05)	< 0.001	3.38 (2.02, 5.66)	< 0.001
P for trend		< 0.001		< 0.001		< 0.001
**Prediabetes**						
Per SD increment	1.35 (1.02, 1.79)	0.035	1.44 (1.10, 1.89)	0.009	1.39 (1.04, 1.84)	0.024
ByG Tertiles						
T1	Reference		Reference		Reference	
T2	1.08 (0.48, 2.46)	0.852	1.13 (0.49, 2.56)	0.778	1.11 (0.48, 2.59)	0.801
T3	2.66 (1.33, 5.34)	0.006	3.06 (1.51, 6.20)	0.002	2.95 (1.44, 6.06)	0.003
P for trend		0.003		< 0.001		0.002

Model 1: adjusted for age, sex and ethnicity. Model 2: adjusted for variables in model 1 plus drinking status, baseline CHD, baseline stroke, smoking status, DBP, and SBP. Model 3: adjusted for variables in model 2 plus ALT, eGFR, TG, HDL-C, ACEIs/ARBs, β-blockers, CCBs, diuretics, statins, AHI, nadir SaO_2_, mean SaO_2_, and regular CPAP treatment

HR, hazard ratio; CI, confidence interval. Other abbreviations appear in Table 2

**Fig. 3:**
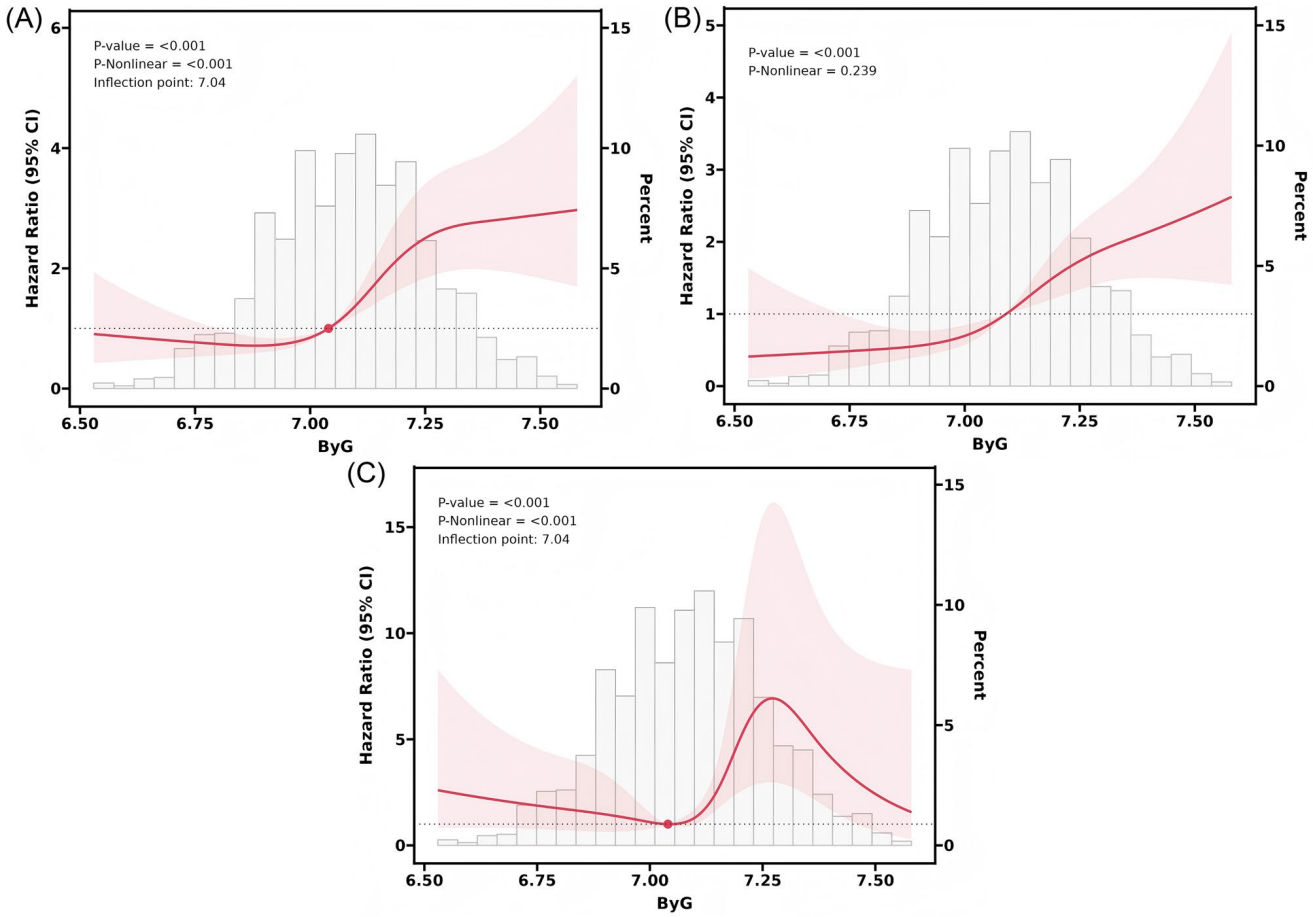
Association between ByG and dysglycemia (**A**), diabetes (**B**), and prediabetes (**C**) using the restricted cubic spline function. Legend: the solid lines represent the HRs and the shaded areas represent the 95% confidence intervals, estimated using restricted cubic splines. The reference value (HR = 1.0) was set at the median of the ByG index. Models were fully adjusted for all covariates listed in Model 3 of the Methods section

Figure 3 shows that the restricted cubic spline analysis revealed significant non-linear relationship between the ByG index and the risks of dysglycemia (Fig. 3A) and prediabetes (Fig. 3C) (P-nonlinear < 0.001). In contrast, the risk of diabetes showed a linear relationship (Fig. 3B, P-nonlinear = 0.239). Significant changes in the risk of dysglycemia and prediabetes were observed when the ByG index was approximately 7.04. Segmented Cox regression analysis at this inflection point showed that the risk of dysglycemia was lower for the ByG index below 7.04, yet significantly elevated at 7.04 and above (Supplemental Table 2). However, the risk of prediabetes remained statistically unchanged before and after the inflection point (Supplemental Table 3).

### Comparison of the ByG index and traditional indicators for early prediction capability

Time-dependent receiver operating characteristic curve analyses were conducted to assess the predictive ability for new-onset dysglycemia, diabetes, and prediabetes. The results showed that while the overall predictive capacity of all indices was modest, the ByG index exhibited the highest AUC at all time points (3, 5, and 7 years) for all three outcomes. For example, in predicting 7-year new-onset dysglycemia, the AUC was 0.672 for the ByG index, compared to 0.604 for TyG-BMI, 0.591 for BMI, 0.578 for WHtR, 0.574 for TyG, and 0.559 for WC. (Supplementary Figs. 1–3).

### Sensitivity analysis

Results remained consistent after sensitivity analyses excluding current smokers (Supplementary Table 4) and alcohol drinkers (Supplementary Table 5). Excluding patients receiving OSA therapy (Supplemental Table 6), participants who developed dysglycemia (both diabetic and prediabetic) within the first year of follow-up (Supplemental Table 7), those using diuretics (Supplemental Table 8), and those with a history of stroke (Supplemental Table 9) did not show significant changes, suggesting that the study results were robust.

### Subgroup analysis

**Fig. 4:**
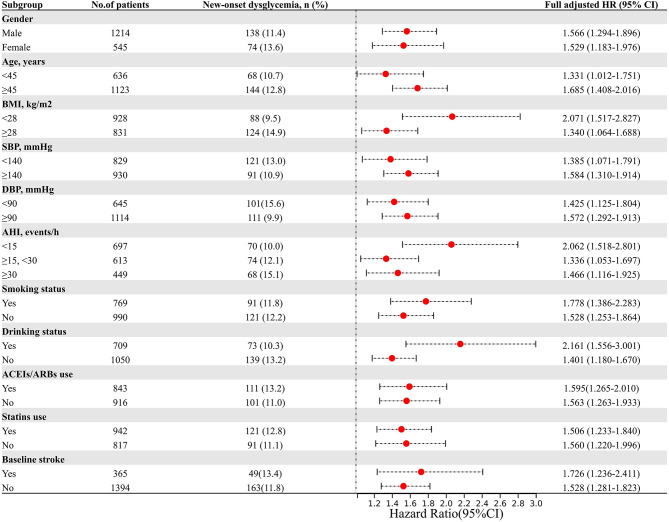

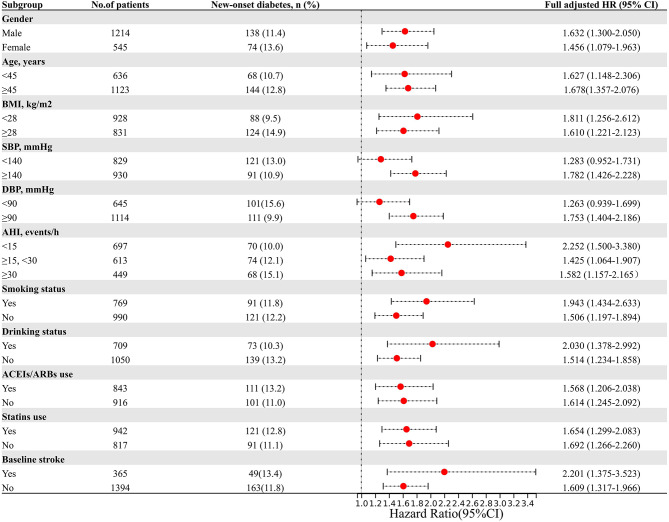

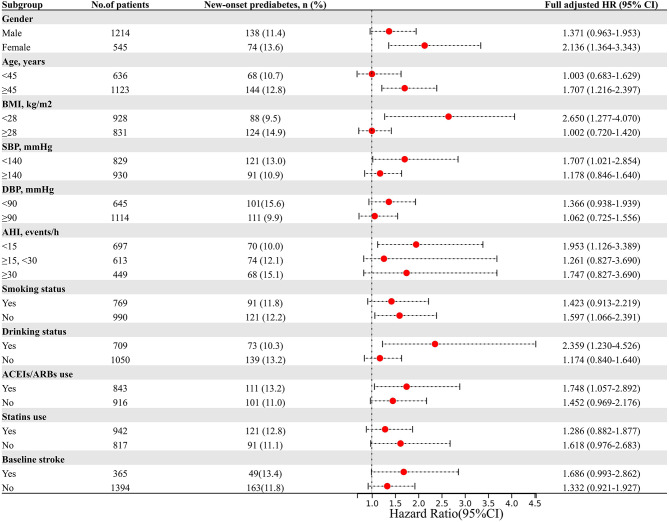
Subgroup analysis of the association between the ByG index and the risk of new-onset dysglycemia (**A**), diabetes (**B**), and prediabetes (**C**). Legend: HRs are presented per one SD increment in the ByG index. All HRs were derived from the fully adjusted Cox model (Model 3). P for interaction was calculated to assess for effect modification across subgroups

Subgroup analysis was conducted to further explore the association between ByG index and the incidence of new-onset dysglycemia (Fig. 4A), diabetes (Fig. 4B), and prediabetes (Fig. 4C). The stratification variables included gender, age, BMI, blood pressure parameters (SBP and DBP), AHI, smoking status, drinking status, and use of statins and ACEIs/ARBs. The results showed that the association between ByG index and dysglycemia was consistent across various subgroups (Supplemental Tables 10–12).

## Discussion

The present retrospective cohort study found a significant association between the ByG index and the risk of new-onset dysglycemia (including diabetes and prediabetes) in patients with hypertension and OSA. Furthermore, the ByG index demonstrated superior early predictive capability for dysglycemia (including diabetes and prediabetes) compared to traditional predictive indicators. This simple and practical tool aids in the early identification of high-risk populations and provides personalized preventive measures for glycemic abnormalities.

Hypertension and OSA tend to coexist, with a prevalence of OSA ranging from 30% to 50% in hypertensive patients [24] and a prevalence of hypertension ranging from 59% to 67% in patients with OSA [25]. This unique subgroup, characterized by the intertwining of both conditions and a high prevalence rate, presents a complex metabolic foundation that significantly increases the risk of glucose abnormalities [7, 8, 26, 27]. However, limited research has addressed the early identification of abnormal glucose metabolism risk in this population. A previous study indicated that the non-dipping blood pressure pattern during 24-hour ambulatory blood pressure monitoring is a risk factor for the onset of diabetes in patients with hypertension and OSA, providing a novel approach for the management of diabetes [23]. However, due to the complexity of diabetes and the multitude of risk factors, relying solely on changes in nighttime blood pressure patterns may not effectively identify high-risk subgroups of diabetes. Moreover, the cost-effectiveness of the widespread application of ambulatory blood pressure monitoring hinders its utility and dissemination for risk assessment. Our research findings demonstrate a strong association between the ByG index, which integrates BMI and blood glucose, and the risk of new-onset of dysglycemia (including diabetes and prediabetes), highlighting the effectiveness of ByG as an early risk predictor. This offers a practical, cost-effective, and widely applicable method to identify individuals at high risk for dysglycemia with both hypertension and OSA, facilitating the development of personalized prevention and management strategies.

The ByG index was initially validated for its association with diabetes risk in a Japanese community-based cohort [16], and no further studies have been conducted to reveal its association with abnormal glucose metabolism. The present study investigated the association between the index and new-onset dysglycemia in hypertensive patients with OSA, confirming the strong association between the index and new-onset diabetes, and further finding its efficacy in identifying the risk of prediabetes. Our study broadens the application of the ByG index for predicting diabetes in various populations and stages. We also uncovered a non-linear correlation between the ByG index and the new-onset of prediabetes and dysglycemia. Once the ByG index surpassed 7.04, the risk of dysglycemia escalated significantly. Intriguingly, we observed a counterintuitive reduction in the risk of prediabetes when the ByG index exceeded 7.27. This phenomenon may be ascribed to the temporal progression from prediabetes to diabetes, with a heightened risk of diabetes development associated with prolonged follow-up and higher ByG index values. This trend may be reversed in a sufficiently large sample size.

While the modest AUC values of the ByG index reflect the challenge of predicting a complex disease like dysglycemia with a single marker [28, 29], its ability to discriminate risk was statistically superior to the other indices tested, highlighting its potential for risk stratification. Furthermore, our Cox regression models confirmed the ByG index as a robust and independent predictor of dysglycemia after extensive multivariable adjustment. These findings suggest that the ByG index, is a simple and effective tool for identifying higher-risk individuals who may warrant closer monitoring and preventive interventions.

A counterintuitive phenomenon was observed in the baseline characteristics of this study: the stroke prevalence at baseline was significantly lower in the highest ByG index tertile group than in the lowest group. Concurrently, this group exhibited the highest age and the lowest proportion of alcohol consumption. It is hypothesised that this may primarily result from individuals with a history of stroke receiving more rigorous secondary prevention and more stringent lifestyle interventions, which may have reduced declines in BMI or fasting blood glucose levels. Consequently, these individuals were more likely to be classified into lower ByG groups at baseline. In order to systematically evaluate the impact of this baseline difference on the study’s core conclusions, a multi-tiered validation was implemented: Firstly, baseline stroke was adjusted as a key covariate in all multivariate Cox models. Secondly, a sensitivity analysis was conducted by excluding all individuals with baseline stroke. Subsequently, a subgroup analysis was performed based on the presence or absence of baseline stroke, and interaction effects were tested. The findings obtained from the study demonstrated that ByG exhibited a consistent association with the onset of dysglycemia, diabetes, and prediabetes. This association was found to be robust and statistically significant (P interaction > 0.05). This finding suggests that baseline differences did not significantly confound the primary outcomes. This finding suggests a potential clinical implication: even in individuals with a history of stroke, the risk of new-onset glucose metabolism abnormalities may be reduced when BMI and fasting blood glucose levels are effectively controlled. While there is biological plausibility to this inference [30, 31], further validation is required through prospective studies and intervention trials.

The efficacy of ByG metrics in detecting the risk of dysglycemia may be attributable to the interplay between hypertension, obstructive sleep apnea (OSA), and obesity [32, 33]. Obesity is commonly associated with OSA in individuals with hypertension, resulting in endothelial dysfunction and increased sympathetic activity that impede glucose uptake [27]. Intermittent nocturnal hypoxia and oxidative stress exacerbate metabolic stress and impair insulin signalling [34]. The integration of BMI and fasting glucose levels in the ByG metric has been shown to summarise the synergistic metabolic effects of obesity, hypertension, and OSA [12, 31, 35, 36]. As expected, this index is valuable for early prediction of the risk of glucose abnormalities in this high-risk population.

In this study, the long-term follow-up with clearly defined cohorts enhanced the credibility of the results. Comprehensive baseline clinical and laboratory data were beneficial for adjusting potential confounding factors, thereby improving the robustness of the findings. Nevertheless, several limitations should be considered. First, the retrospective design is subject to inherent biases, such as selection bias, and limits the ability to establish causality. Second, as a single-center study in Urumqi, China, the generalizability of our findings to populations with different genetic, or lifestyle backgrounds is limited; multi-center studies are required to confirm the applicability of the ByG index in more diverse settings. Third, despite adjusting for numerous covariates, residual confounding by unmeasured variables—such as dietary patterns, physical activity levels, or genetic predisposition—cannot be fully excluded. Fourth, the ByG index has not been externally validated in an independent, prospective cohort. Its clinical utility must be definitively confirmed in future prospective studies before being recommended for clinical screening.

## Conclusion

The ByG index serves as an independent predictor of dysglycemia, encompassing diabetes and prediabetes, among patients with hypertension and OSA. As a simple, accessible, and reliable tool for early risk stratification, it demonstrates application potential but requires validation through prospective studies.

## Supplementary Information

Below is the link to the electronic supplementary material.

Supplementary Material 1

## Data Availability

The datasets used and/or analysed during the current study are available from the corresponding author on reasonable request.

## Abbreviations

ACEIs: Angiotensin-converting enzyme inhibitors
AHI: Apnea-hypopnea index
ARBs: Angiotensin receptor blockers
BMI: Body mass index
ByG: Body mass index-glucose index
CCBs: Calcium channel blockers
CI: Confidence intervals
CPAP: Continuous positive airway pressure
DBP: Diastolic blood pressure
FPG: Fasting plasma glucose
HDL: High-density lipoprotein
OSA: Obstructive sleep apnea
SaO2: Oxygen saturation
SBP: Systolic blood pressure
SD: Standard deviation
TyG: Triglyceride glucose
TyG-BMI: Triglyceride glucose-body mass index
UROSAH: Urumqi Research on Sleep Apnea and Hypertension
WC: Waist circumference
WHtR: Waist-to-height ratio
