# Supplementary material for: The body mass index-glucose index as a new tool for early detection of the risk of dysglycemia in patients with hypertension and obstructive sleep apnea
Source: BMC Endocr Disord. 2026 Mar 19;26:123. doi: 10.1186/s12902-026-02226-w (PMC13122913; doi:10.1186/s12902-026-02226-w)
Supplement: Supplementary file 1 — Supplementary Material 1 [file 12902_2026_2226_MOESM1_ESM.docx]

**Supplemental Material**

**Supplementary Methods:**

**The Details of Polysomnography**

The study was conducted in an accredited sleep laboratory. In the sleep lab, the sleep schedule was regulated, with a “lights-out” time of 12:00 pm and a “lights-on” time of 8:00 am. Participants were not permitted to sleep before lights-out time or sleep past lights-on time. Polysomnography (PSG) consisted of electroencephalograms (C4-A1, O2-A1), right and left electrooculograms, electrocardiogram (ECG), submental and anterior tibial electromyograms, thoracic and abdominal respiratory movements, pulse oxygen saturation, end-tidal and transcutaneous carbon dioxide, oronasal airflow, and nasal pressure. Studies were recorded on an E-series Sleep System (Compumedics, Melbourne, VIC, Australia) and included digital video and audio monitoring.

Studies were scored at a centralized sleep reading center (People’s Hospital of Xinjiang Uygur Autonomous Region, Urumqi, Xinjiang, China) by registered polysomnographic technologists. Sleep and arousals were staged using standard criteria ^1^. Sleep stages were scored using 30-s epochs according to Rechtschaffen and Kales criteria ^2, 3^, and sleep architecture variables were noted as percent total sleep time for stages NREM 1, NREM 2, NREM 3, and REM sleep. Arousals were scored according to published guidelines and summarized as the total number of arousals per hour of sleep (arousal index) ^4^.

To enable the calculation of alternative definitions of the apnea-hypopnea index (AHI), respiratory events were initially identified by changes in signal amplitude only. Specifically, apneas were identified as complete reductions in the thermocouple signal for ≥10 seconds and hypopneas as approximately 30% reductions in the nasal pressure or inductance signals for ≥10 seconds. Each event was annotated as to its associated oxygen saturation nadir and whether it resulted in an EEG arousal.

Definitions of respiratory events.

| Term | Definition |
| --- | --- |
| Apnea | Apnoea was defined as a 90% decrease in airflow lasting for at least 10 seconds. |
| Hypopnea | Hypopneas were defined by ≥ 30% decrement in the amplitude of nasal pressure flow signal for at least 10 seconds, associated with a 4% arterial oxygen desaturation (with or without arousal). |
| AHI | Apnea-hypopnea index = the number of apneas and hypopneas per hours of sleep |

**References**

1. Berry RB, Budhiraja R, Gottlieb DJ, et al. Rules for scoring respiratory events in sleep: update of the 2007 AASM Manual for the Scoring of Sleep and Associated Events. Deliberations of the Sleep Apnea Definitions Task Force of the American Academy of Sleep Medicine. *J Clin Sleep Med*. 2012;8:597-619.

2. Rechtschaffen A, Kales A (1968) A manual of standardized terminology, techniques and scoring system for sleep stages of human subjects. National Institutes of Health, Washington DC.

3. Iber C, Ancoli-Israel S, Chesson A (2007) The AASM manual for the scoring of sleep and associated events: rules, terminology, and technical specifications, 1st edn. American Academy of Sleep Medicine, Westchester.

4. Amerian Academy of Sleep Medicine (1992). EEG arousals: scoring rules and examples: a preliminary report from the Sleep Disorders Atlas Task Force of the American Sleep Disorders Association. Sleep, 15(2), 173-84.

**Supplemental Table 1. Stepwise collinearity diagnostics for model covariates.**

|  | VIF (Step 1) | VIF (Step 2) |
| --- | --- | --- |
| ByG | 30.883 | 1.152 |
| Age | 1.816 | 1.705 |
| Gender | 1.897 | 1.889 |
| Ethnicity | 1.112 | 1.089 |
| BMI | 14.272 | NA |
| Baseline Stroke | 1.149 | 1.149 |
| Baseline CHD | 1.154 | 1.153 |
| Smoking status | 1.728 | 1.727 |
| Drinking status | 1.626 | 1.622 |
| SBP | 2.311 | 2.309 |
| DBP | 2.474 | 2.469 |
| ACEIs/ARBs use | 1.099 | 1.094 |
| Beta blockers use | 1.076 | 1.076 |
| CCBs use | 1.147 | 1.141 |
| Diuretics use | 1.100 | 1.096 |
| Statins use | 1.266 | 1.266 |
| Fasting plasma glucose | 16.276 | NA |
| ALT | 1.157 | 1.156 |
| eGFR | 1.085 | 1.083 |
| Total cholesterol | 2.024 | 2.024 |
| Triglyceride | 1.37 | 1.364 |
| HDL-C | 1.379 | 1.355 |
| LDL-C | 1.805 | 1.805 |
| AHI | 1.513 | 1.501 |
| Mean SaO_2_ | 1.422 | 1.408 |
| Nadir SaO_2_ | 1.707 | 1.695 |
| Regular CPAP treatment (yes vs no) | 1.033 | 1.033 |
| Abbreviations: VIF, variance inflation factor. A stepwise screening method was used: the VIF for each variable was calculated, and the variable with the highest VIF was removed if its value was ≥5. This process was repeated until all remaining variables had a VIF <5. Other abbreviations are defined in Table 1. | | |

**Supplemental Table 2. Cox regression analysis of dysglycemia risk , segmented by a ByG index inflection point of 7.04.**

| Characteristic | HR | 95% CI | p-value |
| --- | --- | --- | --- |
| ByG (< 7.04) | 0.44 | 0.07, 2.65 | 0.369 |
| ByG (≥ 7.04) | 6.26 | 2.10, 17.70 | <0.001 |
| HRs were adjusted for age, sex, ethnicity, drinking status, baseline CHD, baseline stroke, smoking status, DBP, SBP, ALT, eGFR, TG, HDL-C, ACEIs/ARBs, β-blockers, CCBs, diuretics, statins, AHI, nadir SaO2, mean SaO2, and regular CPAP treatment. HR, hazard ratio; CI, confidence interval. Other abbreviations are defined in Table 1. | | | |

**Supplemental Table 3. Segmented cox regression analysis of prediabetes risk based on ByG index with an inflection point at 7.04.**

| Characteristic | HR | 95% CI | p-value |
| --- | --- | --- | --- |
| ByG (< 7.04) | 0.20 | 0.01, 2.64 | 0.15 |
| ByG (≥ 7.04) | 5.23 | 0.60, 47.69 | 0.11 |
| HRs were adjusted for age, sex, ethnicity, drinking status, baseline CHD, baseline stroke, smoking status, DBP, SBP, ALT, eGFR, TG, HDL-C, ACEIs/ARBs, β-blockers, CCBs, diuretics, statins, AHI, nadir SaO_2_, mean SaO_2_, and regular CPAP treatment. HR, hazard ratio; CI, confidence interval. Other abbreviations are defined in Table 1. | | | |

**Supplemental Table 4. Hazard ratios (95% CI) for dysglycemia, diabetes, and prediabetes stratified by ByG index, excluding current smokers**.

| Exposure | Model 1 | | Model 2 | | Model 3 | |
| --- | --- | --- | --- | --- | --- | --- |
|  | (HR, 95% CI) | P-value | (HR, 95% CI) | P-value | (HR, 95% CI) | P-value |
| Dysglycemia |  |  |  |  |  |  |
| Per SD increment | 1.62 (1.34, 1.97) | <0.001 | 1.71 (1.41, 2.09) | <0.001 | 1.57 (1.27, 1.93) | <0.001 |
| Tertiles |  |  |  |  |  |  |
| T1 | Reference |  | Reference |  | Reference |  |
| T2 | 2.20 (1.24, 3.92) | 0.007 | 2.11 (1.19, 3.76) | 0.011 | 1.97 (1.09, 3.54) | 0.024 |
| T3 | 3.45 (2.00, 5.93) | <0.001 | 3.63 (2.10, 6.30) | <0.001 | 2.96 (1.68, 5.22) | <0.001 |
| P for trend |  | <0.001 |  | 0.001 |  | <0.001 |
| Diabetes |  |  |  |  |  |  |
| Per SD increment | 1.66 (1.33, 2.08) | <0.001 | 1.72 (1.37, 2.16) | <0.001 | 1.54 (1.21, 1.97) | <0.001 |
| Tertiles |  |  |  |  |  |  |
| T1 | Reference |  | Reference |  | Reference |  |
| T2 | 2.81 (1.41, 5.60) | 0.003 | 2.71 (1.36, 5.40) | 0.005 | 2.50 (1.23, 5.06) | 0.011 |
| T3 | 3.70 (1.90, 7.22) | <0.001 | 3.75 (1.91, 7.36) | <0.001 | 2.92 (1.45, 5.86) | 0.003 |
| P for trend |  | <0.001 |  | <0.001 |  | 0.004 |
| Prediabetes |  |  |  |  |  |  |
| Per SD increment | 1.49 (1.02, 2.19) | 0.041 | 1.66 (1.12, 2.47) | 0.013 | 1.64 (1.07, 2.52) | 0.023 |
| Tertiles |  |  |  |  |  |  |
| T1 | Reference |  | Reference |  | Reference |  |
| T2 | 1.06 (0.34, 3.30) | 0.922 | 1.01 (0.33, 3.16) | 0.981 | 1.02 (0.32, 3.26) | 0.977 |
| T3 | 2.96 (1.15, 7.61) | 0.024 | 3.52(1.35, 9.18) | 0.010 | 3.40 (1.24, 9.38) | 0.018 |
| P for trend |  | 0.013 |  | 0.005 |  | 0.010 |

Model 1: adjusted for age, sex, and ethnicity.

Model 2: adjusted for variables in model 1 plus drinking status, baseline CHD, baseline stroke, DBP, and SBP.

Model 3: adjusted for variables in model 2 plus ALT, eGFR, TG, HDL-C, ACEIs/ARBs, β-blockers, CCBs, diuretics, statins, AHI, nadir SaO2, mean SaO2, and regular CPAP treatment.

HR, hazard ratio; CI, confidence interval. Other abbreviations appear in Table 1.

**Supplemental Table 5. Hazard ratios (95% CI) for dysglycemia, diabetes, and prediabetes stratified by ByG index, excluding current drinkers**.

| Exposure | Model 1 | | Model 2 | | Model 3 | |
| --- | --- | --- | --- | --- | --- | --- |
|  | (HR, 95% CI) | P-value | (HR, 95% CI) | P-value | (HR, 95% CI) | P-value |
| Dysglycemia |  |  |  |  |  |  |
| Per SD increment | 1.58 (1.33, 1.88) | <0.001 | 1.60 (1.34, 1.91) | <0.001 | 1.45 (1.20, 1.75) | <0.001 |
| Tertiles |  |  |  |  |  |  |
| T1 | Reference |  | Reference |  | Reference |  |
| T2 | 1.97 (1.12, 3.45) | 0.018 | 1.85 (1.05, 3.25) | 0.032 | 1.68 (0.94, 2.98) | 0.078 |
| T3 | 3.82 (2.28, 6.42) | <0.001 | 3.83 (2.26, 6.48) | <0.001 | 3.04 (1.77, 5.22) | <0.001 |
| P for trend |  | <0.001 |  | <0.001 |  | <0.001 |
| Diabetes |  |  |  |  |  |  |
| Per SD increment | 1.74 (1.42, 2.12) | <0.001 | 1.74 (1.42, 2.14) | <0.001 | 1.57 (1.26, 1.96) | <0.001 |
| Tertiles |  |  |  |  |  |  |
| T1 | Reference |  | Reference |  | Reference |  |
| T2 | 2.89 (1.42, 5.91) | 0.004 | 2.69 (1.32, 5.51) | 0.007 | 2.29 (1.11, 4.74) | 0.025 |
| T3 | 5.26 (2.67, 10.34) | <0.001 | 4.92 (2.48, 9.74) | <0.001 | 3.71 (1.84, 7.48) | <0.001 |
| P for trend |  | <0.001 |  | <0.001 |  | <0.001 |
| Prediabetes |  |  |  |  |  |  |
| Per SD increment | 1.17 (0.83, 1.66) | 0.375 | 1.26 (0.89, 1.79) | 0.029 | 1.19 (0.83, 1.71) | 0.348 |
| Tertiles |  |  |  |  |  |  |
| T1 | Reference |  | Reference |  | Reference |  |
| T2 | 0.81 (0.29, 2.25) | 0.692 | 0.82 (0.30, 2.27) | 0.699 | 0.95 (0.33, 2.73) | 0.924 |
| T3 | 2.02 (0.87, 4.68) | 0.103 | 2.55(1.07, 6.09) | 0.035 | 2.39 (0.97, 5.87) | 0.057 |
| P for trend |  | 0.066 |  | 0.023 |  | 0.037 |

Model 1: adjusted for age, sex, and ethnicity.

Model 2: adjusted for variables in model 1 plus, baseline CHD, baseline stroke, smoking status, DBP and SBP.

Model 3: adjusted for variables in model 2 plus ALT, eGFR, TG, HDL-C, ACEIs/ARBs, β-blockers, CCBs, diuretics, statins, AHI, nadir SaO2, mean SaO2, and regular CPAP treatment.

HR, hazard ratio; CI, confidence interval. Other abbreviations appear in Table 1.

**Supplemental Table 6. Hazard ratios (95% CI) for dysglycemia, diabetes, and prediabetes stratified by ByG index,** **excluding patients on OSA therapy.**

| Exposure | Model 1 | | Model 2 | | Model 3 | |
| --- | --- | --- | --- | --- | --- | --- |
|  | (HR, 95% CI) | P-value | (HR, 95% CI) | P-value | (HR, 95% CI) | P-value |
| Dysglycemia |  |  |  |  |  |  |
| Per SD increment | 1.57 (1.36, 1.80) | <0.001 | 1.61 (1.40, 1.86) | <0.001 | 1.53 (1.32, 1.78) | <0.001 |
| Tertiles |  |  |  |  |  |  |
| T1 | Reference |  | Reference |  | Reference |  |
| T2 | 1.76 (1.14, 2.73) | 0.012 | 1.73 (1.11, 2.68) | 0.014 | 1.64 (1.05, 2.56) | 0.029 |
| T3 | 3.36 (2.24, 4.97) | <0.001 | 3.43 (2.30, 5.11) | <0.001 | 3.07 (2.03, 4.63) | <0.001 |
| P for trend |  | <0.001 |  | <0.001 |  | <0.001 |
| Diabetes |  |  |  |  |  |  |
| Per SD increment | 1.65 (1.40, 1.95) | <0.001 | 1.68 (1.43, 1.99) | <0.001 | 1.60 (1.34, 1.91) | <0.001 |
| Tertiles |  |  |  |  |  |  |
| T1 | Reference |  | Reference |  | Reference |  |
| T2 | 2.26 (1.34, 3.81) | 0.002 | 2.25 (1.34, 3.78) | 0.002 | 2.13 (1.26, 3.61) | 0.005 |
| T3 | 3.68 (2.26, 5.98) | <0.001 | 3.75 (2.30, 6.12) | <0.001 | 3.31 (2.00, 5.49) | <0.001 |
| P for trend |  | <0.001 |  | <0.001 |  | <0.001 |
| Prediabetes |  |  |  |  |  |  |
| Per SD increment | 1.35 (1.03, 1.78) | 0.031 | 1.43 (1.09, 1.87) | <0.001 | 1.37 (1.03, 1.81) | 0.028 |
| Tertiles |  |  |  |  |  |  |
| T1 | Reference |  | Reference |  | Reference |  |
| T2 | 0.83 (0.34, 2.01) | 0.684 | 0.83 (0.34, 2.01) | 0.684 | 0.78 (0.32, 1.91) | 0.586 |
| T3 | 2.70 (1.34, 5.39) | 0.005 | 3.11 (1.54, 6.26) | 0.002 | 2.82 (1.37, 5.78) | 0.005 |
| P for trend |  | 0.001 |  | <0.001 |  | 0.001 |

Model 1: adjusted for age, sex, and ethnicity.

Model 2: adjusted for variables in model 1 plus drinking status, baseline CHD, baseline stroke, smoking status, DBP, and SBP.

Model 3: adjusted for variables in model 2 plus ALT, eGFR, TG, HDL-C, ACEIs/ARBs, β-blockers, CCBs, diuretics, statins, AHI, nadir SaO2 and mean SaO2.

HR, hazard ratio; CI, confidence interval. Other abbreviations appear in Table 1.

**Supplemental Table 7. Hazard ratios (95% CI) for dysglycemia, diabetes, and prediabetes stratified by ByG index, excluding participants with dysglycemia occurring within the first year of the follow-up.**

| Exposure | Model 1 | | Model 2 | | Model 3 | |
| --- | --- | --- | --- | --- | --- | --- |
|  | (HR, 95% CI) | P-value | (HR, 95% CI) | P-value | (HR, 95% CI) | P-value |
| Dysglycemia |  |  |  |  |  |  |
| Per SD increment | 1.58 (1.37, 1.82) | <0.001 | 1.63 (1.42, 1.87) | <0.001 | 1.55 (1.33, 1.79) | <0.001 |
| Tertiles |  |  |  |  |  |  |
| T1 | Reference |  | Reference |  | Reference |  |
| T2 | 1.73 (1.11, 2.68) | 0.015 | 1.71 (1.10, 2.66) | 0.016 | 1.62 (1.04, 2.53) | 0.034 |
| T3 | 3.42 (2.38, 5.08) | <0.001 | 3.61 (2.42, 5.37) | <0.001 | 3.19 (2.12, 4.81) | <0.001 |
| P for trend |  | <0.001 |  | <0.001 |  | <0.001 |
| Diabetes |  |  |  |  |  |  |
| Per SD increment | 1.67 (1.42, 1.97) | <0.001 | 1.71 (1.45, 2.02) | <0.001 | 1.62 (1.36, 1.94) | <0.001 |
| Tertiles |  |  |  |  |  |  |
| T1 | Reference |  | Reference |  | Reference |  |
| T2 | 2.21 (1.31, 3.73) | 0.003 | 2.20 (1.30, 3.70) | 0.003 | 2.10 (1.24, 3.56) | 0.006 |
| T3 | 3.82 (2.35, 6.20) | <0.001 | 3.90 (2.40, 6.35) | <0.001 | 3.40 (2.06, 5.63) | <0.001 |
| P for trend |  | <0.001 |  | <0.001 |  | <0.001 |
| Prediabetes |  |  |  |  |  |  |
| Per SD increment | 1.35 (1.02, 1.77) | 0.033 | 1.43 (1.09, 1.87) | 0.009 | 1.37 (1.03, 1.81) | 0.029 |
| Tertiles |  |  |  |  |  |  |
| T1 | Reference |  | Reference |  | Reference |  |
| T2 | 0.82 (0.34, 1.99) | 0.665 | 0.82 (0.34, 1.99) | 0.665 | 0.78 (0.32, 1.90) | 0.577 |
| T3 | 2.65 (1.33, 5.30) | 0.006 | 3.05 (1.52, 6.14) | 0.002 | 2.78 (1.35, 5.70) | 0.005 |
| P for trend |  | 0.002 |  | <0.001 |  | 0.001 |

Model 1: adjusted for age, sex, and ethnicity.

Model 2: adjusted for variables in model 1 plus drinking status, baseline CHD, baseline stroke, smoking status, DBP, and SBP.

Model 3: adjusted for variables in model 2 plus ALT, eGFR, TG, HDL-C, ACEIs/ARBs, β-blockers, CCBs, diuretics, statins, AHI, nadir SaO2, mean SaO2, and regular CPAP treatment.

HR, hazard ratio; CI, confidence interval. Other abbreviations appear in Table 1.

**Supplemental Table 8. Hazard ratios (95% CI) for dysglycemia, diabetes, and prediabetes stratified by ByG index, excluding participants who use diuretics.**

| Exposure | Model 1 | | Model 2 | | Model 3 | |
| --- | --- | --- | --- | --- | --- | --- |
|  | (HR, 95% CI) | P-value | (HR, 95% CI) | P-value | (HR, 95% CI) | P-value |
| Dysglycemia |  |  |  |  |  |  |
| Per SD increment | 1.55 (1.32, 1.82) | <0.001 | 1.60 (1.36, 1.89) | <0.001 | 1.54 (1.30, 1.82) | <0.001 |
| Tertiles |  |  |  |  |  |  |
| T1 | Reference |  | Reference |  | Reference |  |
| T2 | 1.51 (0.94, 2.42) | 0.088 | 1.55 (0.97, 2.49) | 0.067 | 1.44 (1.02, 2.49) | 0.047 |
| T3 | 3.06 (2.00, 4.66) | <0.001 | 3.21 (2.10, 4.91) | <0.001 | 3.06 (1.97, 4.75) | <0.001 |
| P for trend |  | <0.001 |  | <0.001 |  | <0.001 |
| Diabetes |  |  |  |  |  |  |
| Per SD increment | 1.60 (1.32, 1.93) | <0.001 | 1.63 (1.34, 1.97) | <0.001 | 1.56 (1.28, 1.91) | <0.001 |
| Tertiles |  |  |  |  |  |  |
| T1 | Reference |  | Reference |  | Reference |  |
| T2 | 1.82 (1.05, 3.14) | 0.033 | 1.89 (1.03, 3.26) | 0.023 | 1.87 (1.07, 3.26) | 0.027 |
| T3 | 3.04 (1.89, 5.06) | <0.001 | 3.10 (1.86, 5.17) | <0.001 | 2.91 (1.71, 4.94) | <0.001 |
| P for trend |  | <0.001 |  | <0.001 |  | <0.001 |
| Prediabetes |  |  |  |  |  |  |
| Per SD increment | 1.45 (1.07, 1.96) | 0.017 | 1.54 (1.13, 2.10) | 0.006 | 1.49 (1.08, 2.04) | 0.016 |
| Tertiles |  |  |  |  |  |  |
| T1 | Reference |  | Reference |  | Reference |  |
| T2 | 0.81 (0.30, 2.18) | 0.676 | 0.90 (0.40, 2.29) | 0.689 | 0.92 (0.45, 2.30) | 0.587 |
| T3 | 3.08 (1.44, 6.58) | 0.004 | 3.47 (1.61, 7.46) | <0.001 | 3.47 (1.55, 7.66) | 0.002 |
| P for trend |  | <0.001 |  | <0.001 |  | <0.001 |

Model 1: adjusted for age, sex, and ethnicity.

Model 2: adjusted for variables in model 1 plus drinking status, baseline CHD, baseline stroke, smoking status, DBP, and SBP.

Model 3: adjusted for variables in model 2 plus ALT, eGFR, TG, HDL-C, ACEIs/ARBs, β-blockers, CCBs, statins, AHI, nadir SaO2, mean SaO2, and regular CPAP treatment.

HR, hazard ratio; CI, confidence interval. Other abbreviations appear in Table 1.

**Supplemental Table 9. Hazard ratios (95% CI) for dysglycemia, diabetes, and prediabetes stratified by ByG index, excluding participants who with stoke.**

| Exposure | Model 1 | | Model 2 | | Model 3 | |
| --- | --- | --- | --- | --- | --- | --- |
|  | (HR, 95% CI) | P-value | (HR, 95% CI) | P-value | (HR, 95% CI) | P-value |
| Dysglycemia |  |  |  |  |  |  |
| Per SD increment | 1.54 (1.31, 1.82) | <0.001 | 1.59 (1.34, 1.89) | <0.001 | 1.53 (1.28, 1.83) | <0.001 |
| Tertiles |  |  |  |  |  |  |
| T1 | Reference |  | Reference |  | Reference |  |
| T2 | 1.27 (0.77, 2.09) | 0.356 | 1.26 (0.77, 2.08) | 0.360 | 1.22 (0.74, 2.03) | 0.440 |
| T3 | 2.93 (1.90, 4.53) | <0.001 | 2.94 (1.90, 4.54) | <0.001 | 2.72 (1.73, 4.28) | <0.001 |
| P for trend |  | <0.001 |  | <0.001 |  | <0.001 |
| Diabetes |  |  |  |  |  |  |
| Per SD increment | 1.61 (1.34, 1.94) | <0.001 | 1.65 (1.37, 2.00) | <0.001 | 1.59 (1.30, 1.95) | <0.001 |
| Tertiles |  |  |  |  |  |  |
| T1 | Reference |  | Reference |  | Reference |  |
| T2 | 1.37(0.77, 2.44) | 0.278 | 1.37 (0.77, 2.43) | 0.281 | 1.36 (0.76, 2.45) | 0.296 |
| T3 | 3.25 (1.96, 5.36) | <0.001 | 3.24 (1.96, 5.36) | <0.001 | 3.06 (1.80, 5.18) | <0.001 |
| P for trend |  | <0.001 |  | <0.001 |  | <0.001 |
| Prediabetes |  |  |  |  |  |  |
| Per SD increment | 1.35 (0.95, 1.92) | 0.090 | 1.41 (0.98, 2.02) | 0.063 | 1.33 (0.92, 1.93) | 0.135 |
| Tertiles |  |  |  |  |  |  |
| T1 | Reference |  | Reference |  | Reference |  |
| T2 | 1.03 (0.37, 2.84) | 0.960 | 1.04 (0.38, 2.89) | 0.936 | 0.97 (0.34, 2.73) | 0.947 |
| T3 | 2.15 (0.90, 5.10) | 0.084 | 2.23 (0.93, 5.34) | 0.071 | 2.08 (0.85, 5.10) | 0.108 |
| P for trend |  | 0.051 |  | 0.042 |  | 0.064 |

Model 1: adjusted for age, sex, and ethnicity.

Model 2: adjusted for variables in model 1 plus drinking status, baseline CHD, smoking status, DBP, and SBP.

Model 3: adjusted for variables in model 2 plus ALT, eGFR, TG, HDL-C, ACEIs/ARBs, β-blockers, CCBs, diuretics, statins, AHI, nadir SaO2, mean SaO2, and regular CPAP treatment.

HR, hazard ratio; CI, confidence interval. Other abbreviations appear in Table 1.

**Supplemental Table 10. Subgroup analysis of ByG index and new-onset dysglycemia.**

| Subgroup | N | New-onset  dysglycemia, n (%) | Full adjusted HR (95% CI)  Per SD increment | P | P for interaction |
| --- | --- | --- | --- | --- | --- |
| Gender |  |  |  |  | 0.637 |
| Male | 1214 | 138 (11.4) | 1.566 (1.294-1.896) | <0.001 |  |
| Female | 545 | 74 (13.6) | 1.529 (1.183-1.976) | 0.001 |  |
| Age, years |  |  |  |  | 0.339 |
| <45 | 636 | 68 (10.7) | 1.331 (1.012-1.751) | 0.032 |  |
| ≥45 | 1123 | 144 (12.8) | 1.685 (1.408-2.016) | <0.001 |  |
| BMI, kg/m2 |  |  |  |  | 0.055 |
| <28 | 928 | 88 (9.5) | 2.071 (1.517-2.827) | <0.001 |  |
| ≥28 | 831 | 124 (14.9) | 1.340 (1.064-1.688) | 0.013 |  |
| SBP, mmHg |  |  |  |  | 0.357 |
| <140 | 829 | 121 (13.0) | 1.385 (1.071-1.791) | 0.013 |  |
| ≥140 | 930 | 91 (10.9) | 1.584 (1.310-1.914) | <0.001 |  |
| DBP, mmHg |  |  |  |  | 0.880 |
| <90 | 645 | 101(15.6) | 1.425 (1.125-1.804) | 0.003 |  |
| ≥90 | 1114 | 111 (9.9) | 1.572 (1.292-1.913) | <0.001 |  |
| AHI, events/h |  |  |  |  | 0.379 |
| <15 | 697 | 70 (10.0) | 2.062 (1.518-2.801) | <0.001 |  |
| ≥15, <30 | 613 | 74 (12.1) | 1.336 (1.053-1.697) | 0.017 |  |
| ≥30 | 449 | 68 (15.1) | 1.466 (1.116-1.925) | 0.006 |  |
| Smoking status |  |  |  |  | 0.988 |
| Yes | 769 | 91 (11.8) | 1.778 (1.386-2.283) | <0.001 |  |
| No | 990 | 121 (12.2) | 1.528 (1.253-1.864) | <0.001 |  |
| Drinking status |  |  |  |  | 0.145 |
| Yes | 709 | 73 (10.3) | 2.161 (1.556-3.001) | <0.001 |  |
| No | 1050 | 139 (13.2) | 1.401 (1.180-1.670) | <0.001 |  |
| ACEIs/ARBs use |  |  |  |  | 0.983 |
| Yes | 843 | 111 (13.2) | 1.595(1.265-2.010) | <0.001 |  |
| No | 916 | 101 (11.0) | 1.563 (1.263-1.933) | <0.001 |  |
| Statins use |  |  |  |  | 0.937 |
| Yes | 942 | 121 (12.8) | 1.506 (1.233-1.840) | <0.001 |  |
| No | 817 | 91 (11.1) | 1.560 (1.220-1.996) | <0.001 |  |
| Baseline stroke |  |  |  |  | 0.693 |
| Yes | 365 | 49(13.4) | 1.726 (1.236-2.411) | 0.001 |  |
| No | 1394 | 163(11.8) | 1.528 (1.281-1.823) | <0.001 |  |
| Hazard ratios (HRs) represent the risk per standard deviation (SD) increment in the ByG index and are from the fully adjusted model (Model 3), as described in the Methods section. P for interaction was used to assess for effect modification by the subgroup variable. Abbreviations appear in Table 1. | | | | | |

| **Supplemental Table 11. Subgroup analysis of ByG index and new-onset diabetes.** | | | | | |
| --- | --- | --- | --- | --- | --- |
| Subgroup | N | New-onset  diabetes, n (%) | Full adjusted HR (95% CI)  Per SD increment | P | P for interaction |
| Gender |  |  |  |  | 0.670 |
| Male | 1214 | 138 (11.4) | 1.632 (1.300-2.050) | <0.001 |  |
| Female | 545 | 74 (13.6) | 1.456 (1.079-1.963) | 0.014 |  |
| Age, years |  |  |  |  | 0.142 |
| <45 | 636 | 68 (10.7) | 1.627 (1.148-2.306) | 0.006 |  |
| ≥45 | 1123 | 144 (12.8) | 1.678(1.357-2.076) | <0.001 |  |
| BMI, kg/m2 |  |  |  |  | 0.606 |
| <28 | 928 | 88 (9.5) | 1.811 (1.256-2.612) | 0.001 |  |
| ≥28 | 831 | 124 (14.9) | 1.610 (1.221-2.123) | 0.001 |  |
| SBP, mmHg |  |  |  |  | 0.063 |
| <140 | 829 | 121 (13.0) | 1.283 (0.952-1.731) | 0.102 |  |
| ≥140 | 930 | 91 (10.9) | 1.782 (1.426-2.228) | <0.001 |  |
| DBP, mmHg |  |  |  |  | 0.280 |
| <90 | 645 | 101(15.6) | 1.263 (0.939-1.699) | 0.123 |  |
| ≥90 | 1114 | 111 (9.9) | 1.753 (1.404-2.186) | <0.001 |  |
| AHI, events/h |  |  |  |  | 0.294 |
| <15 | 697 | 70 (10.0) | 2.252 (1.500-3.380) | <0.001 |  |
| ≥15, <30 | 613 | 74 (12.1) | 1.425 (1.064-1.907) | 0.017 |  |
| ≥30 | 449 | 68 (15.1) | 1.582 (1.157-2.165） | 0.004 |  |
| Smoking status |  |  |  |  | 0.987 |
| Yes | 769 | 91 (11.8) | 1.943 (1.434-2.633) | <0.001 |  |
| No | 990 | 121 (12.2) | 1.506 (1.197-1.894) | <0.001 |  |
| Drinking status |  |  |  |  | 0.829 |
| Yes | 709 | 73 (10.3) | 2.030 (1.378-2.992) | <0.001 |  |
| No | 1050 | 139 (13.2) | 1.514 (1.234-1.858) | <0.001 |  |
| ACEIs/ARBs use |  |  |  |  | 0.963 |
| Yes | 843 | 111 (13.2) | 1.568 (1.206-2.038) | <0.001 |  |
| No | 916 | 101 (11.0) | 1.614 (1.245-2.092) | 0.001 |  |
| Statins use |  |  |  |  | 0.646 |
| Yes | 942 | 121 (12.8) | 1.654 (1.299-2.083) | <0.001 |  |
| No | 817 | 91 (11.1) | 1.692 (1.266-2.260) | <0.001 |  |
| Baseline stroke |  |  |  |  | 0.697 |
| Yes | 365 | 49(13.4) | 2.201 (1.375-3.523) | 0.001 |  |
| No | 1394 | 163(11.8) | 1.609 (1.317-1.966) | <0.001 |  |
| Hazard ratios (HRs) represent the risk per standard deviation (SD) increment in the ByG index and are from the fully adjusted model (Model 3), as described in the Methods section. P for interaction was used to assess for effect modification by the subgroup variable. Abbreviations appear in Table 1. | | | | | |

| Supplemental Table 12. Subgroup analysis of ByG index and new-onset prediabetes. | | | | | |
| --- | --- | --- | --- | --- | --- |
| Subgroup | N | New-onset prediabetes, n (%) | Full adjusted HR (95% CI)  Per SD increment | P | P for interaction |
| Gender |  |  |  |  | 0.785 |
| Male | 1214 | 138 (11.4) | 1.371 (0.963-1.953) | 0.080 |  |
| Female | 545 | 74 (13.6) | 2.136 (1.364-3.343) | 0.001 |  |
| Age, years |  |  |  |  | 0.083 |
| <45 | 636 | 68 (10.7) | 1.003 (0.683-1.629) | 0.990 |  |
| ≥45 | 1123 | 144 (12.8) | 1.707 (1.216-2.397) | 0.001 |  |
| BMI, kg/m2 |  |  |  |  | 0.064 |
| <28 | 928 | 88 (9.5) | 2.650 (1.277-4.070) | 0.001 |  |
| ≥28 | 831 | 124 (14.9) | 1.002 (0.720-1.420) | 0.823 |  |
| SBP, mmHg |  |  |  |  | 0.170 |
| <140 | 829 | 121 (13.0) | 1.707 (1.021-2.854) | 0.041 |  |
| ≥140 | 930 | 91 (10.9) | 1.178 (0.846-1.640) | 0.333 |  |
| DBP, mmHg |  |  |  |  | 0.128 |
| <90 | 645 | 101(15.6) | 1.366 (0.938-1.939) | 0.298 |  |
| ≥90 | 1114 | 111 (9.9) | 1.062 (0.725-1.556) | 0.758 |  |
| AHI, events/h |  |  |  |  | 0.879 |
| <15 | 697 | 70 (10.0) | 1.953 (1.126-3.389) | 0.017 |  |
| ≥15, <30 | 613 | 74 (12.1) | 1.261 (0.827-3.690) | 0.328 |  |
| ≥30 | 449 | 68 (15.1) | 1.747 (0.827-3.690) | 0.143 |  |
| Smoking status |  |  |  |  | 0.931 |
| Yes | 769 | 91 (11.8) | 1.423 (0.913-2.219) | 0.120 |  |
| No | 990 | 121 (12.2) | 1.597 (1.066-2.391) | 0.023 |  |
| Drinking status |  |  |  |  | 0.020 |
| Yes | 709 | 73 (10.3) | 2.359 (1.230-4.526) | 0.010 |  |
| No | 1050 | 139 (13.2) | 1.174 (0.840-1.640) | 0.348 |  |
| ACEIs/ARBs use |  |  |  |  | 0.971 |
| Yes | 843 | 111 (13.2) | 1.748 (1.057-2.892) | 0.030 |  |
| No | 916 | 101 (11.0) | 1.452 (0.969-2.176) | 0.071 |  |
| Statins use |  |  |  |  | 0.441 |
| Yes | 942 | 121 (12.8) | 1.286 (0.882-1.877) | 0.191 |  |
| No | 817 | 91 (11.1) | 1.618 (0.976-2.683) | 0.062 |  |
| Baseline stroke |  |  |  |  | 0.872 |
| Yes | 365 | 49(13.4) | 1.686 (0.993-2.862) | 0.053 |  |
| No | 1394 | 163(11.8) | 1.332 (0.921-1.927) | 0.128 |  |

| Hazard ratios (HRs) represent the risk per standard deviation (SD) increment in the ByG index and are from the fully adjusted model (Model 3), as described in the Methods section. P for interaction was used to assess for effect modification by the subgroup variable. Abbreviations appear in Table 1. |
| --- |

**Supplementary Figure1.** Time-dependent ROC curve analysis for predicting new-onset dysglycemia.

| 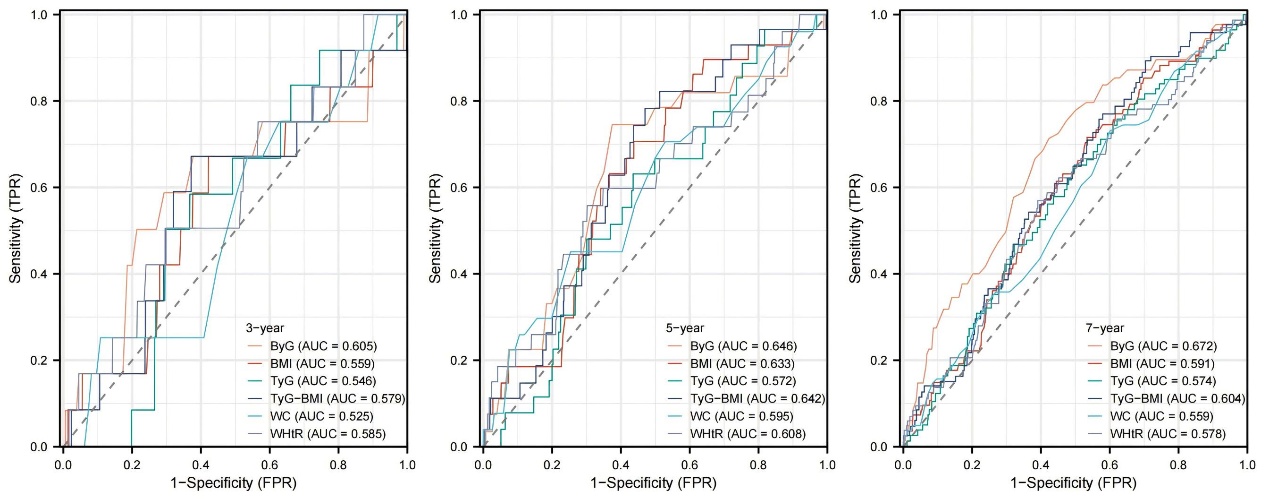 |
| --- |
| Compares the predictive performance of the Body Mass Index(BMI)-Glucose (ByG) index, Triglyceride-glucose (TyG) index, Triglyceride-glucose-body mass index (TyG-BMI), waist circumference (WC), and waist-to-height ratio (WHtR) at 3 years (A), 5 years (B), and 7 years (C). AUC denotes the area under the ROC curve. |

**Supplementary Figure 2** Time-dependent ROC curve analysis for predicting new-onset diabetes.

| 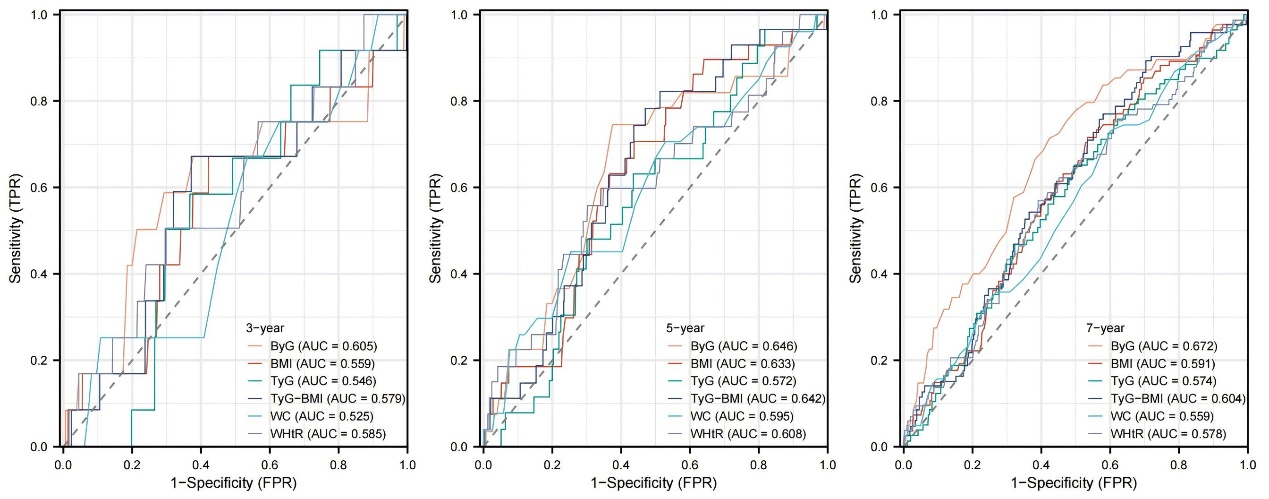 |
| --- |
| Compares the predictive performance of the Body Mass Index(BMI)-Glucose (ByG) index, Triglyceride-glucose (TyG) index, Triglyceride-glucose-body mass index (TyG-BMI), waist circumference (WC), and waist-to-height ratio (WHtR) at 3 years (A), 5 years (B), and 7 years (C). AUC denotes the area under the ROC curve. |

**Supplementary Figure 3** Time-dependent ROC curve analysis for predicting new-onset prediabetes.

| 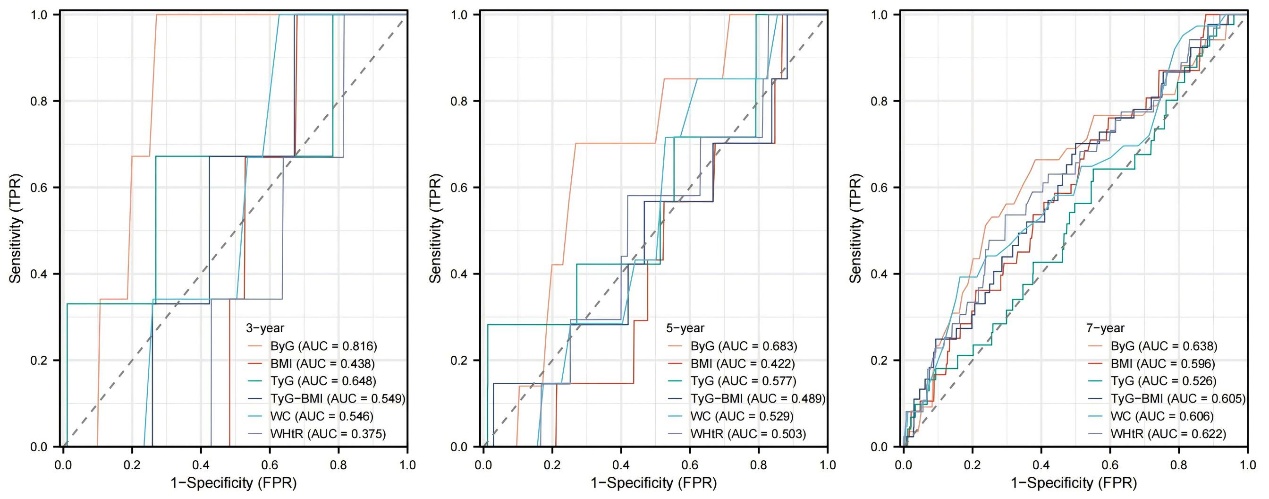 |
| --- |

Compares the predictive performance of the Body Mass Index(BMI)-Glucose (ByG) index, Triglyceride-glucose (TyG) index, Triglyceride-glucose-body mass index (TyG-BMI), waist circumference (WC), and waist-to-height ratio (WHtR) at 3 years (A), 5 years (B), and 7 years (C). AUC denotes the area under the ROC curve.
